# cGAS deficiency enhances inflammasome activation in macrophages and inflammatory pathology in pristane-induced lupus

**DOI:** 10.3389/fimmu.2022.1010764

**Published:** 2022-12-16

**Authors:** Sarinya Kumpunya, Arthid Thim-uam, Chisanu Thumarat, Asada Leelahavanichkul, Nuttiya Kalpongnukul, Naphat Chantaravisoot, Trairak Pisitkun, Prapaporn Pisitkun

**Affiliations:** ^1^ Interdisciplinary Program of Biomedical Sciences, Graduate School, Chulalongkorn University, Bangkok, Thailand; ^2^ Division of Biochemistry, School of Medical Sciences, University of Phayao, Phayao, Thailand; ^3^ Program in Translational Medicine, Faculty of Medicine Ramathibodi Hospital, Mahidol University, Bangkok, Thailand; ^4^ Center of Excellence in Translational Research in Inflammation and Immunology (CETRII), Faculty of Medicine, Chulalongkorn University, Bangkok, Thailand; ^5^ Center of Excellence in Systems Biology, Faculty of Medicine, Chulalongkorn University, Bangkok, Thailand; ^6^ Department of Biochemistry, Faculty of Medicine, Chulalongkorn University, Bangkok, Thailand; ^7^ Epithelial Systems Biology Laboratory, National Heart, Lung and Blood Institute, National Institutes of Health, Bethesda, MD, United States; ^8^ Division of Allergy, Immunology, and Rheumatology, Department of Medicine, Faculty of Medicine Ramathibodi Hospital, Mahidol University, Bangkok, Thailand

**Keywords:** cGAS, inflammasomes, lupus, STING, autoimmune, dsDNA, non-canonical, macrophages

## Abstract

**Introduction:**

Type I interferon (IFN) plays a vital role in the pathogenesis of systemic lupus erythematosus. Cyclic GMP AMP synthase (cGAS) is a cytosolic DNA sensor that recognizes dsDNA and creates cGAMP to activate STING-mediated type I IFN production. The activation of STING induces lupus disease in Fcgr2b deficient mice through the differentiation of dendritic cells. In contrast, Cgas-deficient mice could be generated more autoantibody production and proteinuria in pristane-induced lupus (PIL). These data suggested that the other dsDNA sensors could be involved in lupus development mechanisms.

**Methods:**

This study aimed to identify the cGAS-mediated mechanisms contributing to lupus pathogenesis in PIL. The Cgas-deficient and WT mice were induced lupus disease with pristane and subsequently analyzed autoantibody, histopathology, and immunophenotypes. The lung tissues were analyzed with the expression profiles by RT-PCR and western blot. The bone marrow-derived macrophages were stimulated with inflammasome activators and observed pyroptosis.

**Results:**

The Cgas-/- mice developed more severe pulmonary hemorrhage and autoantibody production than WT mice. The activated dendritic cells, IFN-g-, and IL-17a-producing T helper cells, and infiltrated macrophages in the lung were detected in Cgas-/- mice higher than in WT mice. We observed an increase in expression of Aim2, Casp11, and Ifi16 in the lung and serum IL-1a but IL-1b in pristane-injected Cgas-/- mice. The rise of Caspase-11 in the lung of pristane-injected Cgas-/- mice suggested noncanonical inflammasome activation. The activation of AIM2 and NLRP3 inflammasomes in bone marrow-derived macrophages (BMDMs) enhanced the number of dead cells in Cgas-/- mice compared with WT mice. Activation of the inflammasome significantly induced pyroptosis in Cgas-/- BMDMs. The dsDNA level, but not mitochondrial DNA, increased dramatically in pristane-injected Cgas-/- mice suggesting the dsDNA could be a ligand activating inflammasomes. The cGAS agonist-induced BMDM activation in the Cgas-/- mice indicated that the activation of DNA sensors other than cGAS enhanced activated macrophages.

**Conclusion:**

These findings suggested that cGAS hampers the unusual noncanonical inflammasome activation through other DNA sensors.

## 1 Introduction

Systemic lupus erythematosus (SLE) is an autoimmune disease resulting from loss of tolerance, deposition of immune complexes (ICs), and chronic inflammation. The etiology of SLE derives from various factors, including genetic, environmental, immunoregulatory, hormonal, and epigenetic factors. The heterogeneity of pathogenic mechanisms in mice and human SLE creates complex networks of immune activation. Type I interferon (IFN-I) plays a vital role in SLE pathogenesis. Plasmacytoid dendritic cells (pDCs) are a significant producer of type I IFNs (1). Pattern recognition molecules, including endosomal toll-like receptors (TLRs) 7 and 9, the cytosolic sensor cyclic GMP-AMP synthase (cGAS), and the RNA-sensor RIG-I–like receptor (RLR)-MAVS, can sense nucleic acids, leading to IFN-I production (1). Many cytosolic sensors, such as IFI16, DDX41, and cGAS, sense intracellular double-stranded DNA and subsequently mediate the STING (stimulator of interferon genes) signaling pathway to activate type I IFN production (2, 3).

Cyclic GMP-AMP synthase (cGAS) is a cytosolic DNA sensor that binds with dsDNA in the cytoplasm and synthesizes cyclic guanosine monophosphate–adenosine monophosphate (cyclic GMP-AMP, cGAMP) (4). cGAMP engages STING and recruits TANK-binding kinase 1 (TBK1) to activate IRF3, initiating type I IFN expression (5). Overexpression of cGAS induces robust IRF3 phosphorylation and type I IFN production in a STING-dependent manner (4). cGAMP is transferred from tumor cells to other cells in the tumor microenvironment to activate STING, which induces type I IFNs and triggers NK cell antitumor responses (6). cGAMP derived from tumor cells can be delivered to the cytosolic compartment *via* exosomes to activate the immune response (7).

The STING/cGAS signaling pathway is involved in the pathogenesis of autoimmune diseases. *Trex1*-deficient mice showed severe autoimmune phenotypes, including multiple organ inflammation, autoantibody production, expression of ISGs, and lethality. In contrast, *Trex1/Cgas* double-deficient mice were improved in these autoimmune phenotypes (8) and showed no upregulation of ISG15 and IFN-β (9). Additionally, STING mediates lupus in *Fcgr2b*-deficient mice through the expansion of dendritic cells (10). Moreover, a subgroup of SLE patients increases the expression of *CGAS* in PBMCs and correlates with type I IFN production (11). However, STING showed an inhibitory function against autoimmunity in the MRL. Fas*^lpr^* mice (12), and the absence of STING accelerated collagen-induced arthritis in C57BL/6 mice (13).

Pristane is hydrocarbon oil (2,6,10,14-tetramethylpentadecane, TMPD), which can induce lupus-like phenotypes, including autoantibody production, circulating ICs, glomerulonephritis, and diffuse pulmonary hemorrhage (DPH), when injected into the peritoneal cavity in mice (14, 15). Pristane-induced lupus (PIL) model showed an increase in inflammatory cytokines, including IL-6-, IFN-α-, IFN-ß-, and IFN-γ-induced autoantibodies, in wild-type (WT) mice but not *Ifnar^-/-^* mice (16). Mechanisms of PIL involve different adaptor proteins, such as TRIF (TLRs 3 and 4) and MyD88 (TLRs 7, 8, and 9). Pristane enhances the response of endosomal TLR7 and MyD88, leading to type I IFN and inflammatory cytokine production (14, 17). *Tlr7*-deficient mice have not developed glomerulonephritis or decreased autoantibody production (17). However, *Cgas^-/-^* mice were unprotected from pristane-induced lupus and showed increased autoantibody production, proteinuria, and macrophage expansion (18).

Inflammasomes are a group of multimeric protein sensors in myeloid cells that induce the innate immune response to PAMPs in the cytosol, including AIM2, IFI16, and NLRP3 (8, 19). Several studies have shown that cGAMP activates inflammasomes *via* the AIM2-NLRP3-dependent pathway (20–23). AIM2 is an intracellular DNA sensor that binds to dsDNA and forms the inflammasome complex with the adaptor molecule apoptosis speck-containing protein (ASC) and procaspase-1. These complexes can activate caspase-1 and secrete the proinflammatory cytokines IL-1β and IL-18 (19, 24). The cGAS-STING-NLRP3 axis is activated during viral and bacterial infections in myeloid cells (21). The activation of NLRP3 induces IL-1α secretion and pyroptosis (25). STING/cGAS signaling and the interacting inflammasome complex could contribute to the different phenotypes in autoimmune mouse models. Therefore, the controversial role of the cGAS/STING signaling pathway in the lupus mouse model suggests that further study of the mechanism is needed to verify a potential new therapeutic target for SLE patients.

Here, we found that PIL induced severe lupus activity without cGAS. *Sting* and *Ifi16* upregulated in pristane-injected *Cgas^-/-^* mice which could be influenced by the increased dsDNA. Although IRF3-TBK1 mediated type I IFN was not activated, the expression of Caspase-11 in the lung and serum IL-1α was increased after pristane injection in *Cgas^-/-^* mice. These findings suggested the enhanced noncanonical inflammasome activation by excess dsDNA. Inflammasome activation using the ligands induced more dead cells in *Cgas^-/-^* mice than in WT mice. These data suggested a counterregulatory function between cGAS and the inflammasome signaling pathway.

## 2 Materials and methods

### 2.1 Animals and pristane-induced lupus mouse model

The *Cgas*-deficient mice on the C57BL/6 background were provided by Professor Søren Riis Paludan (Aarhus University, Aarhus, Denmark). WT mice were purchased from Nomura Siam International, Bangkok, Thailand. Eight- to ten-week-old mice were intraperitoneally injected with 500 μl pristane (Sigma) per mouse (16). Eight months after the pristane injection, the mice were sacrificed, and lung, kidney, and spleen tissues and serum samples were collected. All animal experiments were reviewed and approved by the Institutional Animal Care and Use Committees (IACUC) of the Faculty of Medicine, Chulalongkorn University (019/2562 and 024/2563).

### 2.2 Histopathology

Lung and kidney tissues were fixed in 10% neutral buffered formalin for 24 h. Five-micron thick sections of paraffin-embedded (FFPE) tissues were stained with hematoxylin and eosin. The pathology grading from lung and kidney sections was blinded to the analysis by an experienced researcher.

### 2.3 Multiplex immunofluorescence and multispectral imaging

Lung FFPE blocks were cut into three-µm-thick sections and placed on slides. Slide sections were deparaffinized and rehydrated by serial passage through xylene and graded ethanol changes. Slides were subjected to antigen retrieval in AR9 or AR6 buffer (cat. AR9001KT, AR6001KT, Akoya Biosciences) and microwaved at 100% power for 60-90 seconds, then 20% power for 15 minutes. Then, slides were blocked and stained with rabbit monoclonal CD4 (cat. 25229S, clone D7D2Z, 1:400, Cell Signaling), rabbit monoclonal CD8 (cat. 98941S, clone D4W2Z, 1:400, Cell Signaling), rabbit monoclonal CD19 (cat. ab245235, clone EPR23174-145, 1:400, Abcam), rabbit monoclonal F4/80

(cat. ab111101, clone SP115, 1:400, Abcam), and rabbit monoclonal FOX/P3 (cat. 700914, clone 5H10L18, 1:400, Invitrogen) for 30 min at room temperature. The immunofluorescent signal was visualized using the OPAL™ 7-color Manual IHC kit (cat. NEL811001KT, Akoya Biosciences) and counterstained with Spectral DAPI. Then, slides were mounted with ProLongTM diamond antifade (cat. P36961, Invitrogen, CA, USA). All slides were scanned at high-powered 20x magnification using the Vectra Polaris^®^ (Perkin Elmer, MA). Cell phenotyping was analyzed with inForm^®^ Sofware v2.6 (Perkin Elmer, MA).

### 2.4 Measurement of serum autoantibodies

The serum sample was collected eight months after the pristane injection. The anti-nuclear antibodies (ANA) in serum (dilution 1:2000) were detected by indirect immunofluorescence using HEp-2 cells (cat. FA 1512-1010-1, EUROIMMUN, Luebeck, Germany). Samples showed fluorescence intensity and were blindly graded as 4= maximal fluorescence (brilliant yellow-green), 3 = less bright (yellow-green fluorescence), 2= definite (dull yellow-green), and 1= very dim (subdued fluorescence). The levels of autoantibodies (anti-dsDNA) from sera (dilution 1:100) were measured by enzyme-linked immunosorbent assay (ELISA).

### 2.5 Cytokine measurement

Serum cytokine levels were measured using the LEGENDplex™ Mouse Inflammation Panel kit (IL-1α, IL-1β, IL-6, IL-10, IL-12p70, IL-17A, IL-23, IL-27, MCP-1, IFN-β, IFN-γ, TNF-α, and GM-CSF) (cat. 740446, Biolegend, San Diego, CA, USA) according to the manufacturer’s instructions. The beads were analyzed using an LSR II flow cytometer (BD Biosciences), and the data were analyzed by LEGENDplex™ Software version 8 (BioLegend).

### 2.6 Flow cytometry analysis

Single-cell suspensions of 1 x10^6^ splenocytes were stained with flow antibodies, including anti-CD4 (clone: GK1.5; cat. 100423), anti-CD8 (clone: 53-6. 7; cat. 100708), CD3 (clone: 145-2C11; cat. 100312), B220 (clone: RA3-6B2; cat. 103222), CD11c (clone: N418; cat. 117312), IAb (clone: AF6-120.1; cat. 116406), PCDA (clone: 129c1; cat.127103), CD80 (clone: 16-10A1; cat. 104733), F4/80, CD138 (clone: 281-2; cat. 142506), TCR β (clone: H57-597; cat. 109227), and TCR γ/δ (clone: GL3; cat. 118123) cell surface markers (BioLegend, San Diego, CA, USA). For the intracellular cytokine analysis, 1 x10^6^ splenocytes were stimulated with phorbol 12-myristate 13-acetate (PMA), 25 ng/ml, ionomycin 1 µg/ml (Sigma-Aldrich, Darmstadt, Germany), and 1x brefeldin A (BioLegend). Cells were incubated for 4 h at 37°C and stained for anti-CD4, CD8, and CD3 cell surface markers (BioLegend). After permeabilization, the cells were stained for IFN-γ (clone: XMG1. 2: cat. 505821) and IL-17A (clone: TC11-18H10.1: cat. 506921) (BioLegend). Isotype controls were used for each antibody to determine the gates. Flow cytometry was performed using an LSR II flow cytometer (BD Biosciences) and analyzed using FlowJo software version 10 (Tree Star).

### 2.7 Gene expression analysis

Total RNA was extracted from the lung by TRIzol reagent (cat. 15596026, Invitrogen, CA, USA) per the manufacturer’s instructions. RNA was purified using the RNeasy mini kit and treated with DNase I (cat. 74104, Qiagen, MD, USA). Next, 1 µg of total RNA was used as a template for cDNA synthesis using iScript RT Supermix (cat. 1708841, Bio-Rad, California, USA). The expression of genes of interest was assessed by quantitative real-time PCR. The gene expression profiles were tested using SsoAdvanced Universal SYBR Green Supermix (cat. 1725271, Bio-Rad, California, USA). The thermal cycling conditions were as follows: 1 cycle of 95°C for 5 minutes, followed by 40 cycles of 95°C for 15 seconds and 60°C for 1 minute. The relative amounts of target mRNA were normalized to β-actin mRNA as a housekeeping gene and determined by the 2(-ddCt). The primer sequences were as follows:

*Tlr7*: 5’-AATCCACAGGCTCACCCATA-3’, 5’- CAGCTACCAAGGGATGTCCT-3’;

*Tlr9*: 5’-ACTGAGCACCCCTGCTTCTA-3’, 5’- AGATTAGTCAGCGGCAGGAA-3’;

*Rig1*: 5’-GAGAGTCACGGGACCCACT-3’, 5’-CGGTCTTAKCATCTCCAACG-3’;

*Mda5*: 5’-TGATGCACTATTCCAAGAACTAACA-3’, 5’-TCTGTGAGACGAGTTAGCCAAG-3’;

*Sting*: 5’-TCTGACTGTGAGAGCAAGCAG-3’, 5’-ACCTTTAGGTCCCAGGCCATT-3’;

*Ifi16*: 5’-CCAGTCACCAATACTCCACAGC- 3’, 5’- CTCTGAGTGGAGAACAGCACCT-3’

*Ddx41*: 5’-ACAGGAGAAGCGGTTGCCTTTC- 3’, 5’- GACGGCAGTAATACTCCAGGATG-3’

*Dai*: 5’-GATCTACCACTCACGTCAGGAAG- 3’, 5’-GGCAATGGAGATGTGGCTGTTG-3’

*Irf3*: 5’-GCTTGTGATGGTCAAGGTTGT-3’, 5’- AGATGTGCAAGTCCACGGTT-3’;

*Ifnb*: 5’-GCTTGTGATGGTCAAGGTTGT-3’, 5’- AGATGTGCAAGTCCACGGTT-3’;

*Cxcl10*: 5’-CAGTGAGAATGAGGGCCATAGG- 3’, 5’-CGGATTCAGACATCTCTGCTCAT-3’

*Ifng*: 5’-ACTGACTTGAATGTCCAACGCA-3’, 5’- ATCTGACTCCTTTTTCGCTTCC-3’;

*Aim2*: 5’-GGACCCTGTGATGGAGTTG-3’, 5’-GCTCCTTTGTTACCAATCTGATTC-3’;

*Nlrp3*: 5’-GGTCCTCTTTACCATGTGCTTC-3’, 5’-AAGTCATGTGGCTGAAGCTGTA -3’;

*Casp1*: 5’-GGCACATTTCCAGGACTGACTG- 3’, 5’-GCAAGACGTGTACGAGTGGTT G-3’

*Casp11*: 5’-GACGGACCCCAAAAGATGAAG-3, 5’-TGTAGAGTAGAAGGCAATGAAGTC-3’

*Il6*: 5’-TACCACTTCACAAGTCGGAGGC- 3’, 5’-CTGCAAGTGCATCATCGTTGTTC-3’

*Il18*: 5’-GACAGCCTGTGTTCGAGGATATG- 3’, 5’-TGTTCTTACAGGAGAGGGTAGAC-3’

*Il1b*: 5’-GACGGACCCCAAAAGATGAAG-3’, 5’- CTCTTCGTTGATGTGCTGCTGTG-3’

*Actb*: 5’-TAGCACCATGAAGATCAAGAT-3’, 5’-CCGATCCACACAGAGTACTT-3’, and *Il1a*: 5’-TTGAAGACCTAAAGAACTGTTACAGTGAA-3’,5’-GCCATAGCTTGCATCATAGAAGG-3’

### 2.8 *In vitro* bone marrow-derived dendritic cells

BMDCs were obtained from the tibias and femurs. Cells were cultured in RPMI 1640 supplemented with 10% FBS, 1 mM Na pyruvate, 10 mM HEPES buffer, 1% L-glutamine, 1% nonessential amino acids, 100 units/ml pen/strep (Gibco-Thermo Fisher Scientific, MA USA) and 50 μM 2-mercaptoethanol (Sigma-Aldrich, Darmstadt, Germany). Cells were stimulated with 20 ng/ml IL4 and 20 ng/ml GM-CSF (cat. 130-097-757 and 130-095-746, Miltenyi, Bergisch Gladbach, Germany) and maintained at 37°C in a CO2 incubator for 5 days. Immature dendritic cells were transfected with 1 µg/ml cGAS agonist (G3-ended Y-form Short DNA, cat. tlrl-ydna) by LyoVac (cat. lyec-12) reagent and activated by adding 1 µg/ml RIG-1 agonist (5’ppp-dsRNA, cat. tlrl-3prna), 10 μg/ml DMXAA (5,6-dimethylxanthenone-4-acetic acid or STING ligand, cat. tlrl-dmx), and 1 μg/ml TLR7 agonist (Gardiquimod™, cat. tlrl-gdq-5) (Invivogen, San Diego, USA) for 24 hours. Mature BMDCs were stained with the following antibodies: anti-PDCA, anti-CD80, anti-I-Ab, anti-CD11b, and anti-CD11c (BioLegend) and analyzed by flow cytometry (BD Biosciences).

### 2.9 Inflammasome assays

Bone marrow-derived macrophages (BMDMs) were flushed from the tibias and femurs. Cells were cultured in RPMI 1640 supplemented with 10% FBS, 1 mM Na pyruvate, 10 mM HEPES buffer, 1% L-glutamine, 1% nonessential amino acids, 100 units/ml pen/strep (Gibco-Thermo Fisher Scientific, MA USA), 50 μM 2-mercaptoethanol (Sigma-Aldrich, Darmstadt, Germany), and 20 ng/ml recombinant mouse macrophage-colony stimulating factor (M-CSF; cat. 576402 Biolegend, San Diego, CA, USA) for 7 days. Briefly, cells were plated into 24-well plates at 2 × 105 per well in complete RPMI 1640 without M-CSF overnight. Cells were washed once with PBS and primed with ultrapure LPS (200 ng/mL) (cat. tlrl-pb5lps) for 3 h, followed by stimulation with NLRP3 activators (2 µM nigericin) (cat. tlrl-nig), AIM2 activator (1 µg/mL poly(dA:dT) (cat. tlrl-patc), and cGAS agonist (1 µg/ml G3-YSD) (cat. tlrl-ydna) (*In vivo*Gen, San Diego, USA) for 24 hours. After stimulation, the supernatants were collected, and BMDMs were stained with the following antibodies: anti-F4/80, B220, I-Ab, CD11b, and CD11c (Biolegend) and analyzed by flow cytometry (BD Biosciences).

### 2.10 Western blot analysis

Lung tissues were lysed on ice in buffer C containing 1% Triton X-100 and supplemented with a protease inhibitor and phosphatase inhibitor cocktail. Proteins were separated by SDS-PAGE, then proteins were transferred to nitrocellulose membranes and probed with STING antibody (clone: GTN-01; 1:2000) (CUSB-in house, BKK, TH), Phospho-TBK1 antibody (Ser172) (clone D52C2, cat: 5483; 1:1000) (Cell Signaling, MA, USA), TBK1 antibody (clone D1B4, cat: 3504; 1:1000) (Cell Signaling, MA, USA), Caspase11 antibody (clone 17D9, cat: 14340; 1:1000) (Cell Signaling, MA, USA), and Gasdermin D antibody (clone E9S1X, cat: 39754; 1:1000) (Cell Signaling, MA, USA). After incubation at 4°C overnight, the membrane was washed and probed with IRDye^®^ 680RD Donkey anti-Rabbit IgG (H + L) and IRDye^®^ 800CW Donkey anti-Mouse IgG (H + L) secondary antibody (1:10000) (LI-COR, Lincoln, Nebraska, USA) for 1 hour at room temperature. The signals determined the membrane by ODYSSEY CLx (LI-COR, Lincoln, Nebraska, USA). Quantification of the western blot was analyzed by Image studio, and data were normalized against housekeeping proteins.

### 2.11 dsDNA and mitochondrial DNA quantification

One of three spleens was extracted by FavorPrep™ Tissue Genomic DNA Extraction assay (cat. FATGK001-1) (Favorgen Biotech corp, Wembley, WA, Australia). The dsDNA levels were measured using the Qubit™ dsDNA HS Assay Kits (cat. Q32854, Thermo Fisher Scientific, MA USA). For each assay, 1 µl of total DNA was combined with 199 µl of working solution and incubated for 2 min. The Qubit™ 4 Fluorometer was used to quantify the dsDNA levels. Next, mtDNA was measured by quantitative real-time PCR. The gene expression profiles were tested using SsoAdvanced Universal SYBR Green Supermix (cat. 1725271, Bio-Rad, California, USA). The thermal cycling conditions were as follows: 1 cycle of 95°C for 5 minutes, followed by 40 cycles of 95°C for 15 seconds and 60°C for 1 minute. The relative amounts of mitochondrial DNA were normalized to β-2-microglobulin and determined by the 2(-ddCt). The primer sequences were as follows: mMito: 5’-CGTACACCCTCTAACCTAGAGAAGG-3’, 5’- GGTTTTAAGTCTTACGCAATTTCC-3’ and mβ2m: 5’-TTCTGGTGCTTGTCTCACTGA -3’, 5’- CAGTATGTTCGGCTTCCCATTC-3’

### 2.12 Creatinine assays

The serum sample was collected four months after pristane injection. Serum creatinine was determined by QuantiChrom Creatinine-Assay (cat. DICT-500, BioAssay, Hayward, CA, USA).

### 2.13 Statistical analysis

Student’s unpaired t-test was used to calculate two-tailed p values to estimate the statistical significance of differences between two treatment groups using GraphPad Prism software (version 7, San Diego, CA, USA). For all experiments, data will be expressed as the mean ± s.e.m. and p values < 0.05 were considered statistically significant: *p ≤ 0.05 and **p ≤ 0.01.

## 3 Results

### 3.1 Loss of the cGAS sensor promotes autoantibody production and the pulmonary inflammatory response in the pristane-induced lupus model

**Figure 1:**
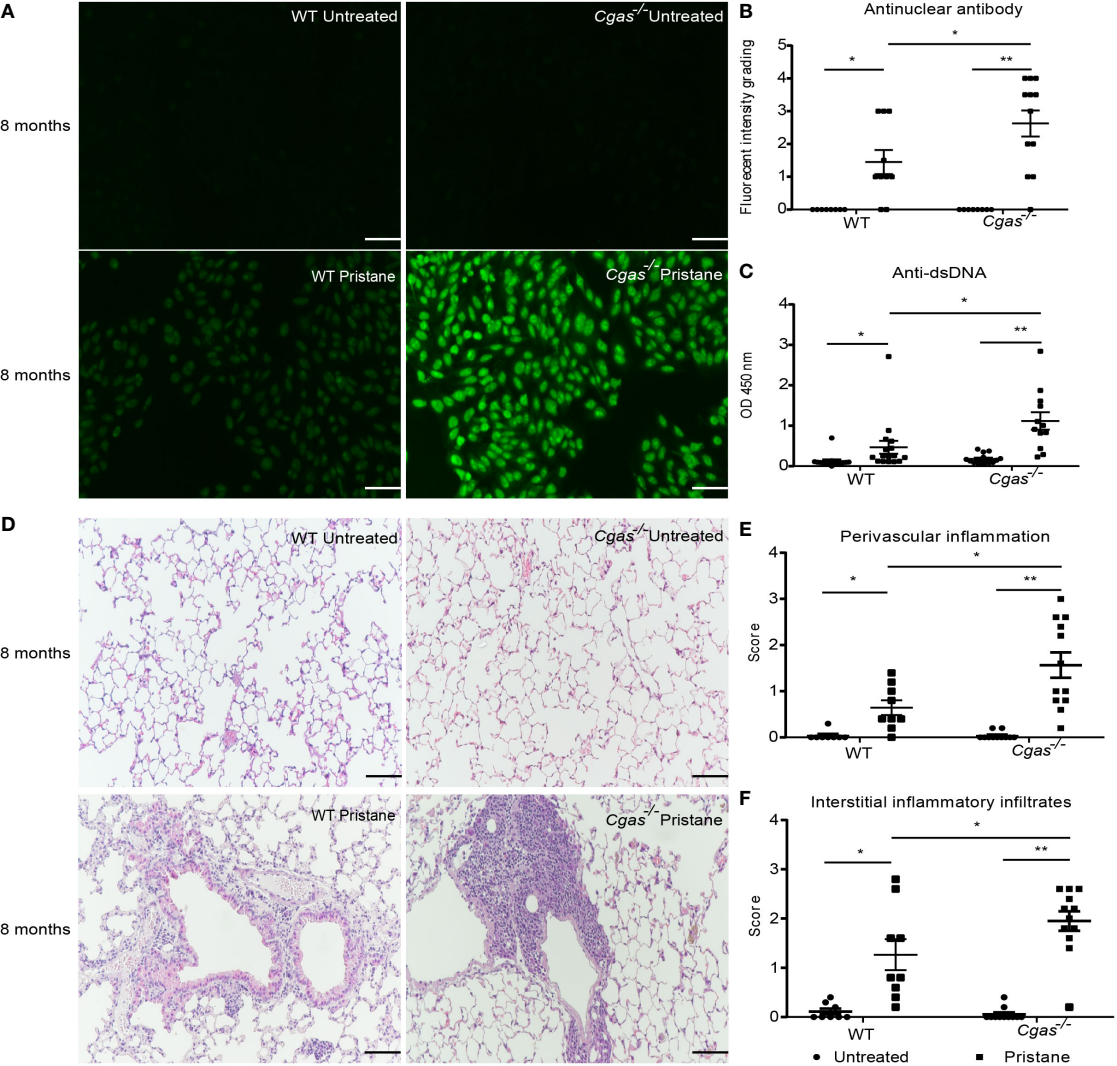
Loss of the cGAS sensor promotes autoantibody production and the pulmonary inflammatory response in the pristane-induced lupus model. The mice were sacrificed at 8 months to collect the tissues and sera after pristane injection. **(A)** The anti-nuclear antibodies (ANA) from sera were detected using HEp2 substrate slides at a 1:2000 dilution (n = 8-12 per group). Representative pictures from each group are shown at 200X magnification (scale bar, 100 µm). **(B)** The slides were graded by fluorescence intensity. **(C)** Serum levels of anti-dsDNA were detected by ELISA (n = 12-16 per group). **(D)** Lung tissues were stained with H&E. Representative pictures from each group are shown at 200X magnification (scale bar, 100 µm). **(E, F)** Perivascular inflammation and interstitial inflammatory infiltrate scores of lung sections were blindly graded (n = 8-12 per group). Data are shown as the mean ± SEM of 3-5 independent experiments **(A–F).** *p < 0.05, **p < 0.01.

The levels of an anti-nuclear antibody (ANA) and anti-dsDNA antibody in the serum from pristane-injected *Cgas^-/-^* mice were significantly higher than those in the serum from pristane-injected WT mice **(** **Figures 1A–C** **).** The lung pathology showed a higher score of perivascular inflammation and interstitial infiltration in the pristane-injected *Cgas^-/-^* mice than in WT mice **(** **Figures 1D–F** **).** In addition, the kidneys of pristane-injected *Cgas^-/-^* mice showed inflammation and enlarged glomeruli. The glomerulonephritis and interstitial nephritis scores in the kidneys of pristane-injected *Cgas^-/-^* mice were significantly higher than those in WT mice. **(** **Supplemental Figures S1A–C** **).** These data suggested that loss of the cGAS sensor promotes autoantibody production, increases inflammatory phenotypes, and aggravates lupus severity in PIL.

### 3.2 cGAS deficiency expands the activated dendritic cells and plasmacytoid dendritic cells in response to pristane

**Figure 2:**
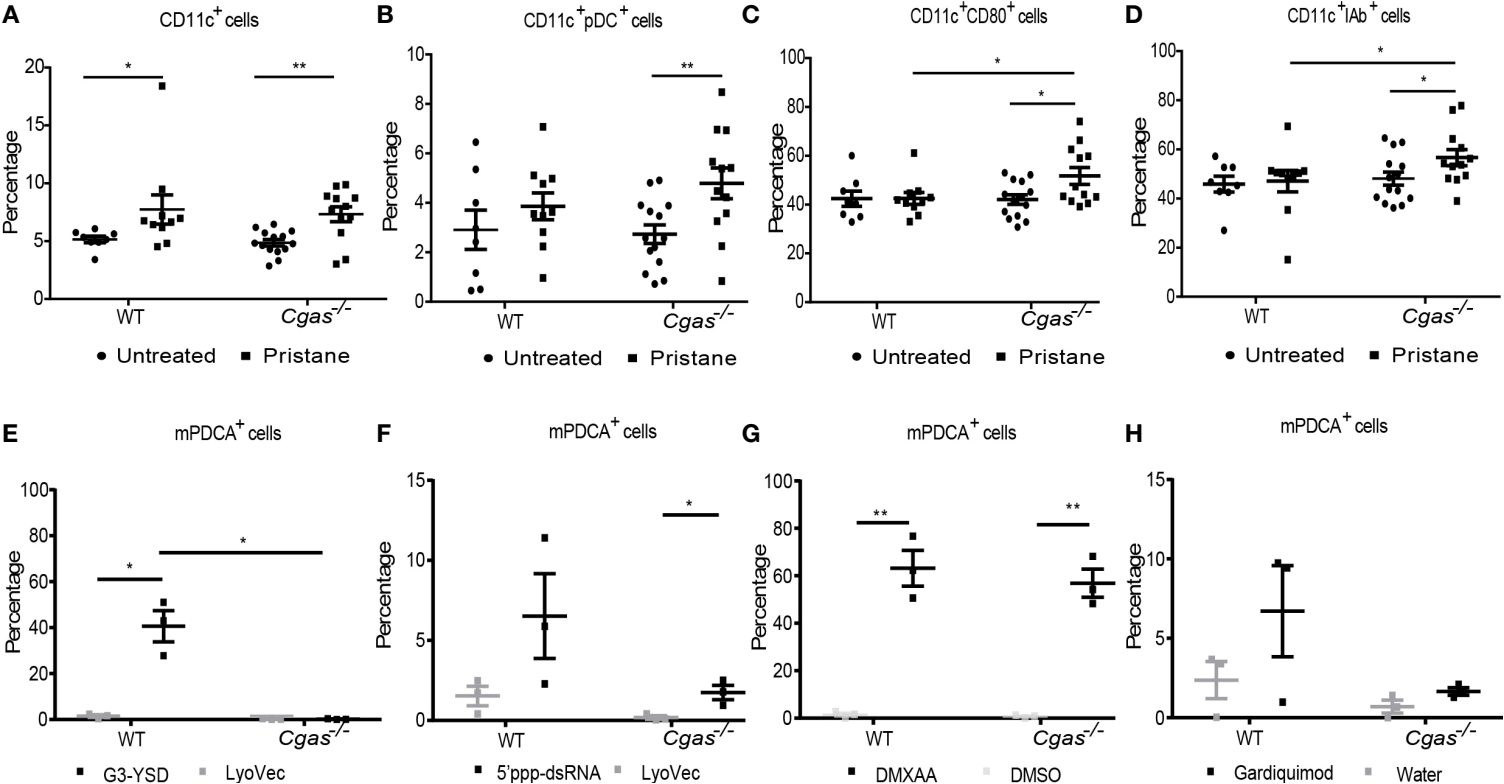
cGAS deficiency expands the activated dendritic cells and plasmacytoid dendritic cells in response to pristine. **(A–D)** Flow cytometry analysis of splenocytes isolated from WT and *Cgas^-/-^* mice at 8 months after pristane injection (n = 8-14 per group). Data are shown as the percentage of **(A)** CD11c^+^ cells, **(B)** CD11c^+^PDCA^+^ (plasmacytoid DC), **(C)** CD11c^+^CD80^+^ cells, and **(D)** CD11c^+^ IAb^+^ cells. **(E–H)** Bone marrow-derived dendritic cells (BMDCs) were differentiated with IL-4 and GM-CSF for 5 days. Flow cytometry analysis shows the percentage of pDCs after **(E)** G3-YSD activation, **(F)** 5’ppp-dsRNA activation, **(G)** DMXAA activation, and **(H)** Gardiquimod activation for 24 h (n = 3 per group). Data are shown as the mean ± SEM. *p < 0.05, **p < 0.01.

Previous work suggests STING activation promotes DC maturation and pDC differentiation in *Fcgr2b*-deficient lupus mice (10). Here, we found that pristane induced the expansion of dendritic cells (CD11c^+^) in both WT and *Cgas^-/-^* mice but only generated a higher number of plasmacytoid dendritic cells (CD11c^+^PDCA^+^) in *Cgas^-/-^* mice (**Figures 2A, B**, **Supplemental Figures S2A, B**). Although the expansion of CD11c^+^ cells did not differ between WT and *Cgas^-/-^* mice, the percentage of activated dendritic cells (CD11c^+^CD80^+^ cells and CD11c^+^IAb^+^) from pristane-injected *Cgas^-/-^* mice was significantly higher than that from pristane-injected WT mice (**Figures 2C, D**). Contrary to STING-induced dendritic cell expansion in *Fcgr2b*-deficient mice, these data suggested that cGAS signaling inhibited the accumulation of pDCs and activated DCs in the pristane-induced lupus model.

Next, we explored whether cGAS activation can promote pDC expansion *in vitro*, similar to STING, and examined whether cGAS signaling could respond to other ligands differently to mediate the differentiation of pDCs. Bone marrow-derived dendritic cells (BMDCs) were differentiated into immature DCs and stimulated with a cGAS agonist, RIG-I agonist, DMXAA, and TLR7 agonist. The *in vitro* culture of BMDCs with cGAS agonist showed a significant increase in pDC cells in the WT mice but not in *Cgas^-/-^* mice (**Figure 2E**). The RIG-I agonist showed a slight rise in pDC cells in the *Cgas^-/-^* mice (**Figure 2F**). Moreover, the immature DCs from WT and *Cgas^-/-^* mice also showed an increasing percentage of pDC cells after DMXAA stimulation (**Figure 2G**). In contrast, the TLR7 agonist showed increased pDC cells in WT mice compared with *Cgas^-/-^* mice (**Figure 2H**). These *in vitro* data suggested that cGAS signaling expands the differentiation of pDCs, similar to STING activation. The *in vitro* data differentiation of pDCs may not be the actual situation in pristane-induced lupus. However, *in vivo* experiments showed the increased pDC expansion in cGAS-deficient mice, which hints to us to investigate the underlying mechanism of these findings that’s more complex in pristane-induced lupus than *in vitro*. The discrepancy between *in vivo* and *in vitro* data suggested the possibility of other DNA sensors that could still signal without cGAS *in vivo*.

### 3.3 The absence of cGAS accelerates the increase of inflammatory T cells and macrophages in pristane-induced lupus

**Figure 3:**
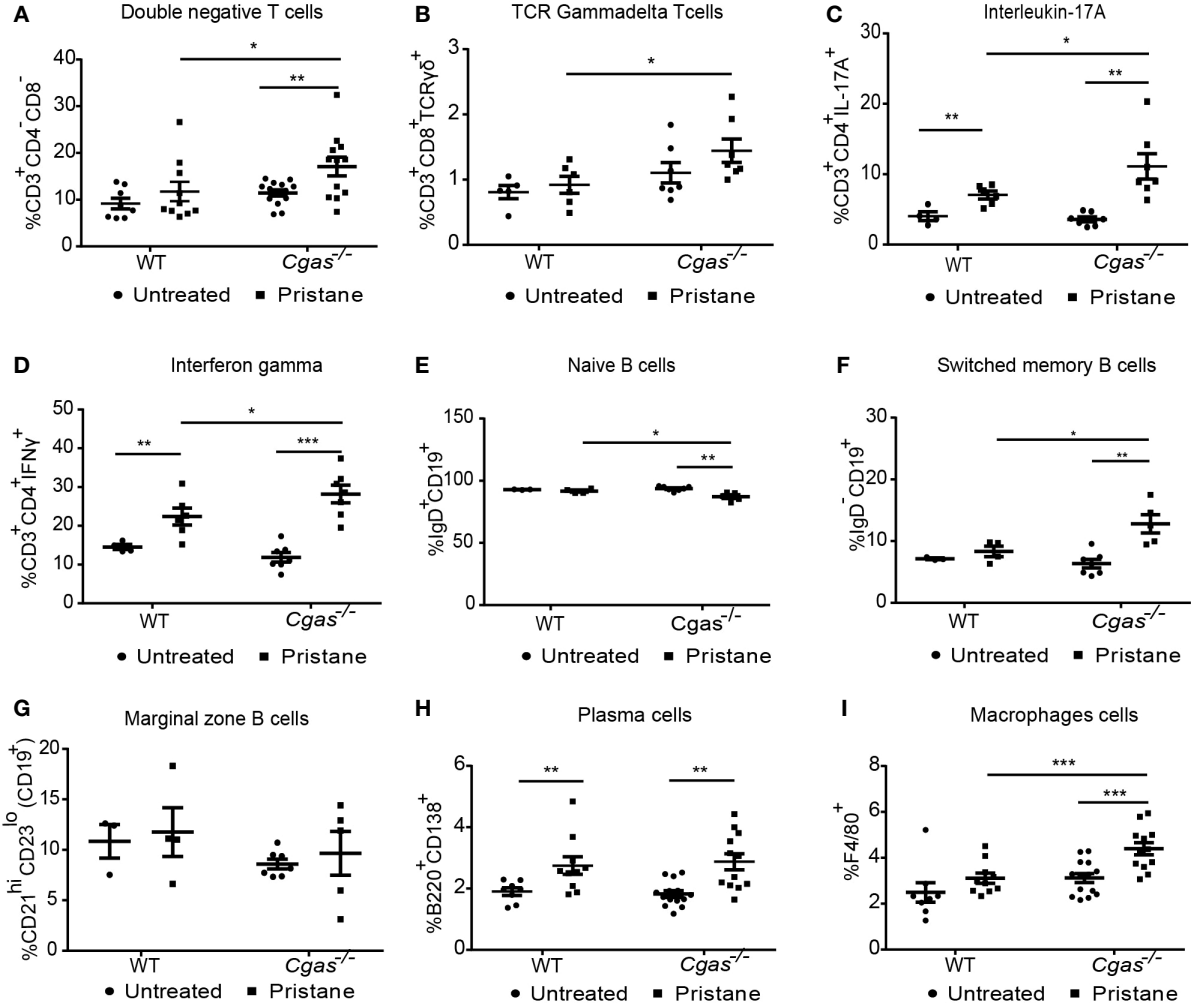
The absence of cGAS accelerates the increase of inflammatory T cells and macrophages in pristane-induced lupus. **(A–I)** Flow cytometry analysis of splenocytes isolated from WT and *Cgas^-/-^* mice at 8 months after pristane injection (n = 8-14 per group). Data are shown as the percentage of **(A)** double-negative T cells (CD3^+^CD4^-^CD8^-^), **(B)** TCR gamma-delta T cells (CD3^+^CD8^+^TCR-γδ^+^), **(C)** interleukin-17A (CD3^+^CD4^+^IL-17A+), **(D)** interferon-gamma (CD3^+^CD4^+^IFN-γ^+^), **(E)** naïve B cells (CD19^+^IgD^+^), **(F)** switched memory B cells (CD19^+^IgD^-^), **(G)** marginal zone B cells (CD19^+^CD21^hi^CD23^low^), **(H)** plasma cells (B220^+^CD138^+^), and **(I)** macrophage cells (F4/80^+^). Data are shown as the mean ± SEM. *p < 0.05, **p < 0.01, *** p < 0.001.

Flow cytometry was used to determine the profile of immune cells in the splenocytes of WT and *Cgas^-/-^* mice. The percentages of double-negative T cells (CD3^+^CD4^-^CD8^-^), TCR gamma-delta T cells (CD3^+^CD8^+^TCRγδ^+^), interleukin-17A (CD3^+^CD4^+^IL-17A^+^), and interferon-gamma (CD3^+^CD4^+^IFN-γ^+^)-producing T helper cells in pristane-injected *Cgas^-/-^* mice were significantly higher than those in pristane-injected WT mice (**Figures 3A–D**). Then we analyzed the B cell population that may be affected by the difference in T cell interaction. We detected the reduction of naïve B cells (CD19^+^IgD^+^) (**Figure 3E**) and the increase in the switched memory B cells (CD19^+^IgD^-^) in *Cgas^-/-^* mice but not WT. While pristane injection could not induce marginal zone B cells (CD19^+^CD21^hi^CD23^low^) expansion (**Figure 3G**), the plasma cells (B220^+^CD138^+^) similarly showed a significant increase in the pristane-injected WT and *Cgas^-/-^* mice (**Figure 3H**). In addition, the percentage of macrophages (F4/80^+^) in pristane-injected *Cgas^-/-^* mice was significantly higher than that in pristane-injected WT mice (**Figure 3I**). These data suggest that cGAS signaling inhibits the expansion of cytokine-producing T helper cells, switched B cells, and macrophages in PIL.

### 3.4 cGAS deficiency promotes the infiltration of macrophages in the lung

**Figure 4:**
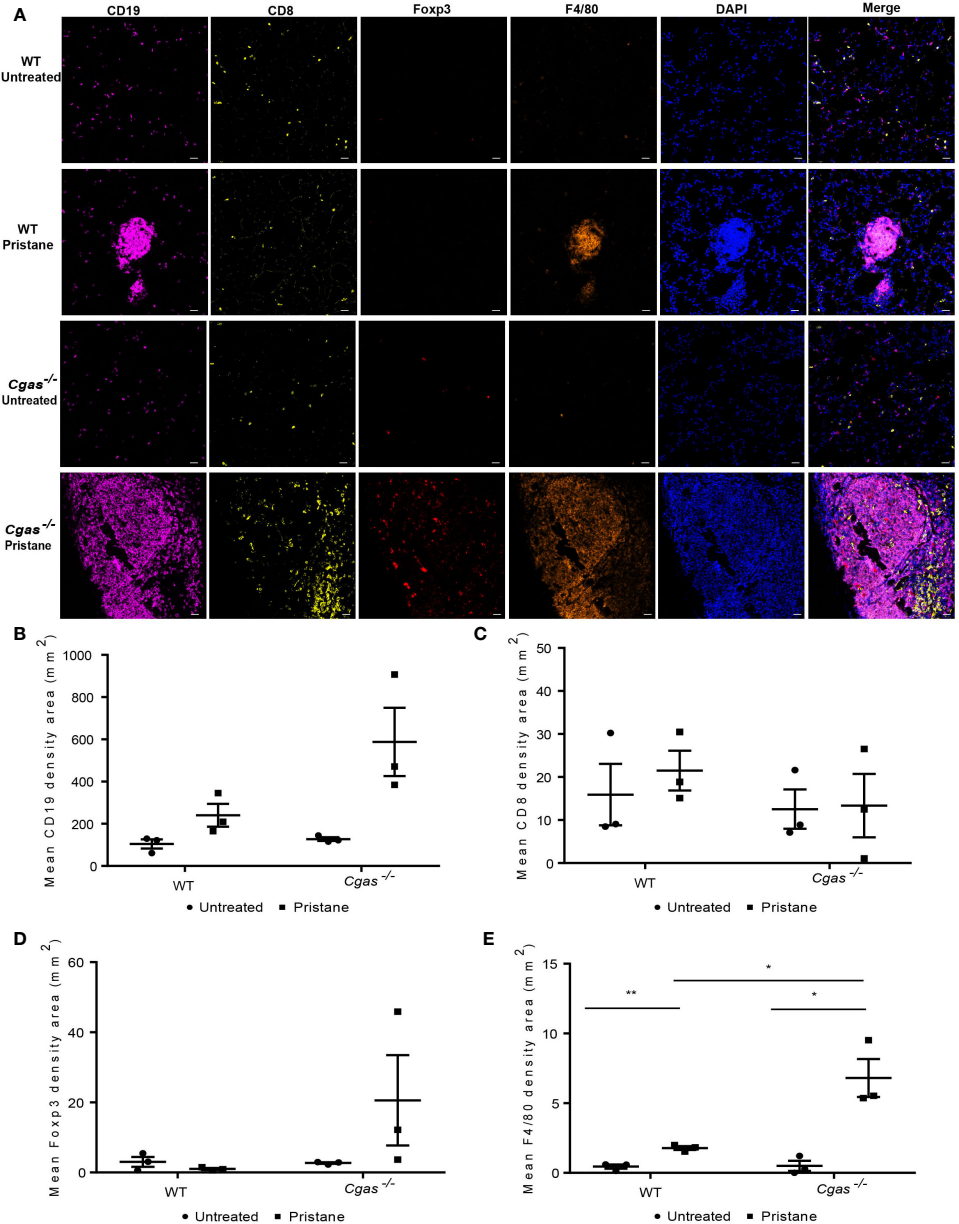
cGAS deficiency promotes the infiltration of macrophages in the lung. **(A)** Multiplex immunofluorescence staining of the lung from WT and *Cgas^-/-^* mice at 8 months after pristane injection shows CD19 (magenta), CD8 (yellow), Foxp3 (red), F4/80 (orange), and DAPI (blue). Data represent 3 mice per group (scale bar, 500 µm). **(B–E)** The mean density area of CD19 **(B)**, CD8 **(C)**, Foxp3 **(D)**, and F4/80 **(E)** (n = 3 per group). Data are shown as the mean ± SEM. *p < 0.05, **p < 0.01.

To assess the expression of immune cells in the lungs of the pristane-induced lupus model, lungs from WT and *Cgas^-/-^* mice were stained with multiplex immunofluorescence at 8 months after pristane injection (**Figure 4A** **).** The infiltration of B cells (CD19^+^) in pristane-injected *Cgas^-/-^* mice showed a higher trend than that in pristane-injected WT mice but did not achieve a significant level **(** **Figure 4B** **).** Also, no difference in T cells (CD8^+^) and Treg cells (Foxp3^+^) were detected **(** **Figures 4C, D** **).** Moreover, the density area of macrophage cells (F4/80^+^) in the pristane-injected *Cgas^-/-^* mice was significantly higher than that in the pristane-injected WT mice **(** **Figure 4E** **).** The data suggested that the proinflammatory cells that promote lung pathology in PIL of the *Cgas^-/-^* mice were macrophages.

### 3.5 Inhibition of cGAS increases the expression of Caspase 11 but not phosphorylation TBK1 in the lungs of pristane-induced lupus mice

**Figure 5:**
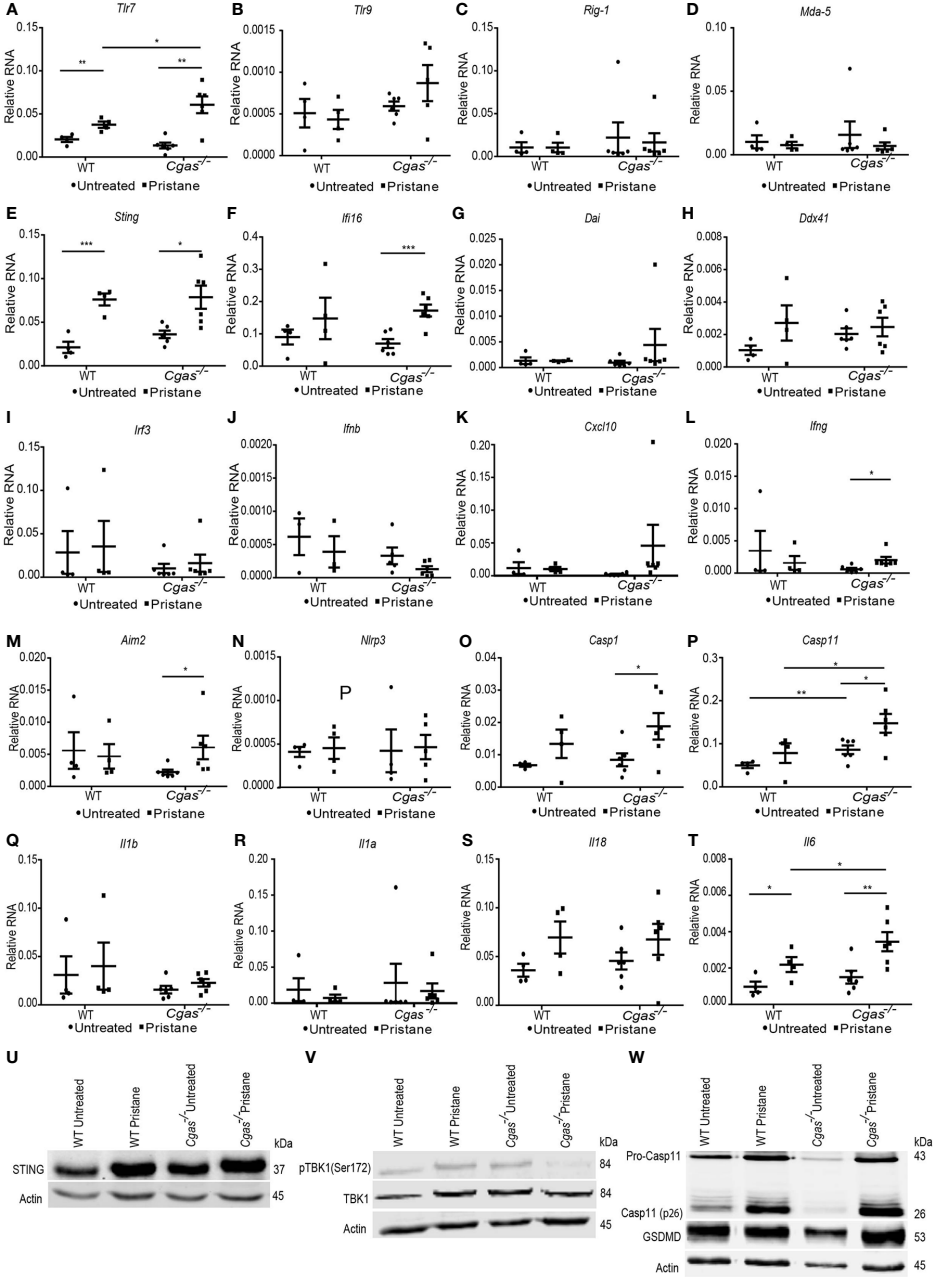
Inhibition of cGAS increases the expression of Caspase 11 but not phosphorylation TBK1 in the lungs of pristane-induced lupus mice. **(A–L)** The relative RNA expression (normalized to actin) of **(A)** *Tlr7*, **(B)** *Tlr9*, **(C)** *Rig1*, **(D)** *Mda5*, **(E)** *Sting*, **(F)** *Ifi16*, **(G)** *Dai*, **(H)** *Ddx41*, **(I)** *Irf3*, **(J)** *Ifnb*, **(K)** *Cxcl10*, **(L)** *Ifng*, **(M)** *Aim2*, **(N)** *Nlrp3*, **(O)** *Casp1*, **(P)** *Casp11*, **(Q)** *Il1b*, **(R)** *Il1a*, **(S)** *Il18, and* **(T)** *Il6* from the lungs of WT and *Cgas^-/-^* mice at 8 months after pristane injection (n = 4-6 per group). **(U–W)** Western blot analysis of **(U)** STING, **(V)** phosphorylation of TBK1 (Ser172), and **(W)** caspase-11 and gasdermin D from the lungs of WT and *Cgas^-/-^* mice at 8 months after pristane injection (representative blot, n = 3). Data are shown as the mean ± SEM. *p < 0.05, **p < 0.01, *** p < 0.001.

Next, we determined which expression levels of nucleic acid sensors and interferon-related genes were increased in PIL. We found that *Tlr7* was significantly more upregulated in pristane-injected *Cgas^-/-^* mice than in pristane-injected WT mice (**Figure 5A**). In contrast, the expression of *Tlr9*, *Rig1*, and *Mda5* was not upregulated in PIL (**Figures 5B–D**). Although *Sting* upregulated in WT and *Cgas^-/-^* pristane injected mice (**Figure 5E**), the expression of *Ifi16* (mouse *Ifi202*) only increased in *Cgas^-/-^* pristane injected mice (**Figure 5F**). The expression of *Dai* and *Ddx41* did not upregulate (**Figures 5G, H** **)**. This data suggested the Ifi16 may be a compensatory function for the lack of cGAS.

*Irf3*, *Ifnb*, and *Cxcl10* were not upregulated in either *Cgas^-/-^* or WT mice (**Figures 5I–K**), suggesting the absence of STING-mediated interferon expression. However, the expression of *Ifng* was significantly upregulated in pristane-injected *Cgas^-/-^* mice compared to cGAS untreated mice (**Figure 5L**). We next tested whether the downstream pathway of STING-mediated type I IFN production was activated in *Cgas^-/-^* mice. The lung tissues of PIL showed increased protein expression of STING in pristane-injected WT and *Cgas^-/-^* mice (**Figure 5U**). However, the phosphorylation of TBK1 showed an increase in pristane-injected WT mice but not *Cgas^-/-^* mice (**Figure 5V**), suggesting the inactivated downstream of STING-mediated type I IFN in the *Cgas^-/-^* mice. These data suggested another signaling pathway that is not the type I interferon signaling pathway that simultaneously activated *Ifng* expression in the absence of cGAS.

In addition to the STING/cGAS signaling pathway, DNA can also stimulate inflammasomes. Thus, we tested the expression of inflammasome molecules and found an increase in *Aim2* expression but not *Nlrp3* in pristane-injected *Cgas^-/-^* mice (**Figures 5M, N** **)**. In addition, the expression of *Casp1* increased in *Cgas^-/-^* pristane-injected mice, and *Casp11* upregulated in the lung of *Cgas^-/-^* mice compared to WT (non-treated and pristane-injected mice) (**Figures 5O, P** **)**. In contrast, the expression of *Il1b*, *Il1a*, and *Il18* was not upregulated in the *Cgas^-/-^* and WT mice (**Figures 5Q–S**). Although *Il6* expression was increased in pristane-injected *Cgas^-/-^* and WT mice, *Cgas^-/-^* mice showed significantly higher *Il6* expression than WT mice (**Figure 5T** **).** Since the appearance of AIM 2 correlates with lupus severity (26), the increase of *Aim2*, *Casp1*, and *Casp11* expression suggested the possibility of inflammasome activation causing pathology of the lungs.

Next, we performed the western blot to confirm the noncanonical inflammasome activation in the pristane-injected lung of *Cgas^-/-^* mice. Pristane induced protein expression of Caspase-11, especially the active Caspase-11 (cleaved form) in the lung of *Cgas^-/-^* mice, more than WT (**Figure 5W**, **Supplemental Figures S3A, B**). In addition, the gasdermin D, downstream of Caspase-11 mediated pyroptosis, showed a trend of increase in the *Cgas^-/-^* mice more than WT (**Figure 5W**, **Supplemental Figure S3C**). The data suggested the activation of noncanonical inflammasomes activation.

### 3.6 The absence of cGAS induces inflammasome activation

**Figure 6:**
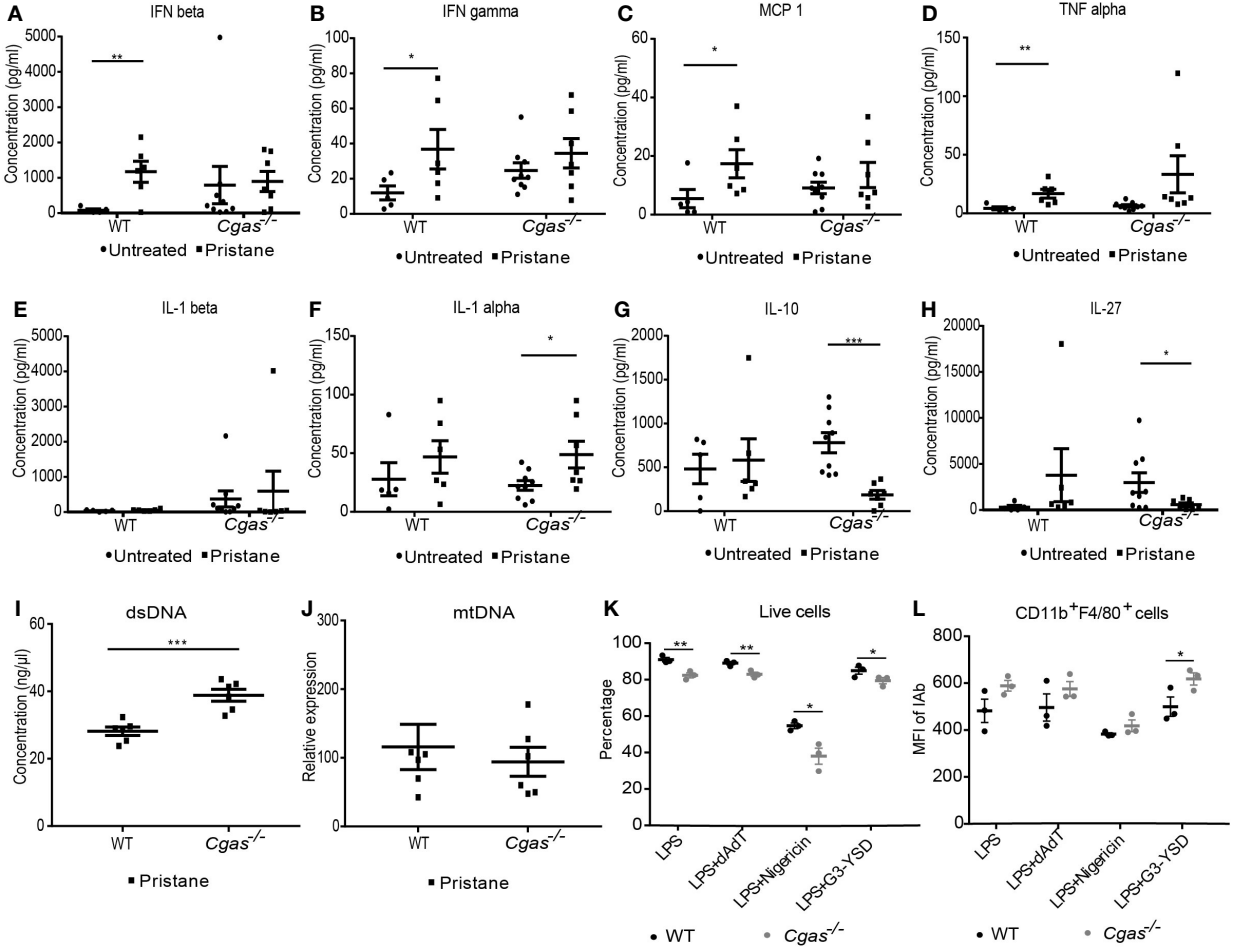
The absence of cGAS induces inflammasome activation. **(A–H)** The cytometric bead array analyzed the serum cytokines of WT and *Cgas^-/-^* mice at 8 months after pristane injection. Serum cytokines **(A)** IFN-β, **(B)** IFN-γ, **(C)** MCP-1, **(D)** TNF-α, **(E)** IL-1β, **(F)** IL-1α, **(G)** IL-10, and **(H)** IL-27 (n = 5-9 per group). **(I)** The dsDNA level from the spleen of WT and *Cgas^-/-^* mice at 8 months after pristane injection (n = 6 per group). **(J)** The Quantitative real-time PCR analysis of mitochondrial DNA (mtDNA) (normalized to β-2-microglobulin) from the spleen of WT and *Cgas^-/-^* mice at 8 months after pristane injection (n = 6 per group). Bone marrow-derived macrophages (BMDMs) were differentiated with M-CSF1 for 7 days. Flow cytometry analysis shows the percentage of live cells **(K)** and the mean fluorescence intensity of CD11b^+^F4/80^+^IAb^+^ cells **(L)** after dAdT, nigericin, and G3-YSD activation for 24 hours (n = 3 mice per group). Data are shown as the mean ± SEM. *p < 0.05, **p < 0.01, *** p < 0.001.

We determined the proinflammatory cytokines in the serum of the pristane-induced lupus model. We found that the serum levels of IFN-β, IFN-γ, MCP-1, and TNF-α in pristane-injected WT mice were significantly increased compared with those in control WT mice but not in *Cgas^-/-^* mice (**Figures 6A–D**). Although the levels of IL-1β did not increase in either WT or *Cgas^-/-^* mice (**Figure 6E**), the serum IL-1α level was significantly increased in pristane-injected *Cgas^-/-^* mice (**Figure 6F**). In contrast, the serum IL-10 and IL-27 levels in pristane-injected *Cgas^-/-^* mice were significantly decreased compared with those in control *Cgas^-/-^* mice (**Figures 6G, H** **)**. These data suggested that *Cgas^-/-^* mice had increased proinflammatory IL-1α levels, indicating inflammasome activation in PIL. To explore the possible ligands that activate the inflammasomes, we tested dsDNA and mitochondrial DNA, which can activate cGAS and inflammasomes (4, 27–29). We detected the increase of dsDNA (**Figure 6I**) but not mitochondrial DNA **(** **Figure 6J** **)** in pristane-injected *Cgas^-/-^* mice. The rise in dsDNA can powerfully activate the inflammasome (22).

Because the inflammatory macrophages detected in the lung were cGAS dependent, we tested macrophage function using bone marrow-derived macrophages (BMDMs) from both WT and *Cgas^-/-^* mice to determine the role of cGAS in inflammasome activation. BMDMs were primed with LPS and stimulated with dAdT (AIM2 ligands), nigericin (Nlrp3 ligands), and a cGAS agonist and analyzed by flow cytometry. Interestingly, *Cgas^-/-^* mice showed a significant decrease in the live cell percentage across all stimulations (**Figure 6K**). In addition, the combination of LPS and nigericin synergistically decreased cell survival in BMDMs (**Figure 6K**). The data suggested that cGAS signaling mediated survival through inflammasome activation in BMDMs. Furthermore, the mean fluorescence intensity of IAb^+^ cells was increased in CD11b^+^F4/80^+^ cells after a G3-YSD (cGAS ligands) in *Cgas^-/-^* mice (**Figure 6L**, **Supplemental Figure S4A**). The IAb expression on macrophages did not prefer to present on high FSC (or enlarged) or high SSC cells (**Supplemental Figures S4B, C**). G3-YSD may enhance the excessive activation of other DNA sensors besides cGAS. Suggesting a noncanonical pathway by which the appropriate cGAS function may be essential to avoid excessive inflammation. These data indicated that cGAS is involved in activating inflammasomes and macrophages in the PIL model. Without cGAS to process the dsDNA, this excess dsDNA could overflow to stimulate inflammasomes.

## 4 Discussion

Cytosolic DNA sensors such as IFI16, DDX41, and cGAS mediate type I IFN production through STING (2, 3). Our previous study showed STING-mediated lupus in a spontaneous mouse model, *Fcgr2b*-deficient mice, through both IFN-I and non-IFN-I signaling pathways (10). However, STING may affect the pathogenesis of other lupus mouse models differently. The pristane-induced lupus model (PIL) has shown lupus-like phenotypes, including autoantibody production, circulating ICs, glomerulonephritis, and diffuse pulmonary hemorrhage (DPH). In addition, pristane injected into the peritoneal cavity produced type I IFN and inflammatory cytokines (14, 15, 30, 31).

A study reported that *Cgas^-/-^* mice were unprotected from pristane-induced SLE (18). In this study, we demonstrated that pristane-induced lupus in *Cgas^-/-^* mice lead to increased autoantibody levels in the serum, inflammatory cell infiltration in the lung, and lupus nephritis. The induction of pDC to produce type I IFN is mediated through other DNA sensors such as TLR9, DDX41, and DHX36 (32–34). The pDC differentiation *in vitro* required STING/cGAS signaling; however, macrophage infiltration in the lung and expansion in the spleen were increased in *Cgas^-/-^* mice, suggesting a specific role of cGAS in macrophages that is different from cGAS function in dendritic cells.

The cGAS-STING pathway can activate human pDCs to produce type I and III IFN (35). We found similarity in the expansion of CD11c^+^ cells from WT and *Cgas^-/-^* mice after PIL. However, pristane-injected *Cgas^-/-^* mice showed higher percentages of CD80^+^ and MHC-II^+^ dendritic cells from the spleen, suggesting that cGAS controls the activation of dendritic cells. Specific B cell populations could have PDCA staining and expanded in lupus (36). The pDC analysis could partly contain this B cell subset; thus, we cannot demonstrate the specific role of pDC in this study. Moreover, we explored the differentiation of pDCs from WT and *Cgas^-/-^* mice. The results showed increased pDC expansion in both WT and *Cgas^-/-^* mice after stimulation with a STING agonist (DMXAA) and RIG-I agonist. The *Cgas^-/-^* mice did not exhibit increased pDC differentiation after cGAS agonist stimulation. These data suggested that activated DCs from *Cgas^-/-^* mice may promote the generation of IFNγ-producing CD4^+^ T cells.

We found increased double-negative T cells, TCRγδ T cells, IL-17A-, and IFNγ-producing T helper cells in pristane-injected *Cgas^-/-^* mice. However, plasma cell expansion showed similarity in both pristane-injected WT and *Cgas^-/-^* mice. These data suggested that inhibition of cGAS signaling activates both type 1 and type 3 immune responses. In addition, the percentage of macrophages increased in pristane-injected *Cgas^-/-^* mice. Therefore, the interaction between macrophages and T cells may increase and aggravate lupus severity. IFNγ-signaling on B cells is required for class-switching autoantibody production (37). The increase in the switched B cells of *Cgas^-/-^* mice after pristane injection could be enhanced by IFNγ-producing T cells.

Pristane-treated C57BL/6 mice develop perivascular inflammation and infiltration of macrophages, neutrophils, lymphocytes, and eosinophils (38, 39). When the inflammation in the lung occurs, the alveoli will be injured, causing alveolar macrophage (AM) damage. Then, circulating monocytes in the capillaries are recruited to the lungs and transformed into AM-like cells (40). The infiltration of macrophages in *Cgas^-/-^* mouse lungs was higher than that in WT mouse lungs after pristane injection. Although the B cell infiltration in the lung of *Cgas^-/-^* mice seems to be increased compared to WT after pristane injection, the stats did not show a significant difference. Thus, we thought a trend of B cell expansion in *Cgas^-/-^* mice could be indirectly promoted by interferon-gamma expression in the lung.

Pristane-induced type I IFN and inflammatory cytokine production may involve the TLR7 and TLR9 signaling pathways (14, 31, 39). We detected high expression of *Tlr7* but not *Tlr9* in the lungs of pristane-injected *Cgas^-/-^* mice. In contrast, we noticed a low expression of *Rig1*, *Mda5*, and *Nlrp3* in both WT and *Cgas^-/-^* mice. The upregulated *Sting* expression in the pristane-injected *Cgas^-/-^* mice could be enhanced by other DNA sensors that still function in the *Cgas^-/-^* mice as we detected the increased *Ifi16* expression in pristane-injected *Cgas^-/-^* mice. The role of the upregulated STING expression could be the compensated mechanism that responds to excess dsDNA and maintain the readiness activity if the cells get the infection. The blunt expression of *Irf3*, *Ifnb*, and *Cxcl10* and lack of phosphorylated TBK1 in the lung and serum IFN-β suggested non-STING-IRF3-TBK mediated IFN-I pathway in the pristane-injected *Cgas^-/-^* mice.

However, the expression of the *Aim2* genes was increased in pristane-injected *Cgas^-/-^* mice. These findings suggested that the absence of cGAS may activate other compensatory mechanisms to increase the signal of cytosolic DNA sensing. STING-mediated type I IFN signaling requires the entire length of STING, while the other function does not (41). IFI16 is necessary for the cGAMP-induced STING activation and interacts with STING to promote STING phosphorylation and translocation. IFI16 and cGAS cooperate in STING activation during DNA sensing (42). To induce type I IFN production through STING-IRF3-TBK activation required both IFI-16 and cGAS. Without cGAS, STING could freely interact with AIM2 and NLRP3 (43, 44), which could induce inflammasome activation. The absence of cGAS increased the expression of both AIM2 and IFI16, which may activate more inflammasomes and lung injury.

The alveolar macrophages in normal mice are anti-inflammatory and express IL-10 (45, 46). The activated alveolar macrophage could secrete proinflammatory cytokines such as IL-6 and TNF-α, leading to enhanced lung injury. Impaired phagocytosis and pyroptosis of alveolar macrophages result in exacerbated lung injury. Thus, the excessive activation of macrophages in the lung could create a devastating outcome in pristane-induced lupus mice (40). IL-6 is a biomarker of severe fatal SARS-CoV2 pneumonia and acute exacerbation of acute interstitial lung diseases (47, 48). IL-6 plays a significant role in severe lung inflammation (49), and blocking IL-6 signaling improves the pulmonary outcome in several conditions (50–52). The increased expression of the proinflammatory cytokine IL-16 in the lung of pristane-injected *Cgas^-/-^* mice may derive from activated inflammatory macrophages and lead to severe lung pathology and lethality. We found that the serum levels of IL-10 decreased in pristane-injected *Cgas^-/-^* mice. IL-27 shows an immunosuppressive effect by promoting T regulatory cell function and inhibiting Th17 differentiation (53). The reduction in IL-10 and IL-27 could contribute to the inflammatory phenotypes in pristane-injected *Cgas^-/-^* mice.

A previous study reported that pristane-injected *Aim2^-/-^* mice developed less kidney inflammation, autoantibody production, and proteinuria (54). Activating NLRP3 and AIM2 canonical inflammasomes activate caspase-1 and secrete the proinflammatory cytokines IL-1β and IL-18 (19, 55, 56). The caspase-1 inflammasome components NLRP3 and NLRC4 are not expressed in lung epithelial cells. Therefore, caspase-11 noncanonical inflammasome activation in these cells only triggers pyroptosis without the processing and release of IL-1β and IL-18 (57). The higher Caspase 11 expression in the lung of *Cgas^-/-^* mice compared to WT suggested the noncanonical inflammasome activation in *Cgas^-/-^* mice. Expression of *Aim2* and *Casp1* was upregulated in *Cgas^-/-^* pristane mice but not WT mice, which may suggest a certain degree of canonical inflammasome activation induced by pristane.

We found that the serum levels of IFN-β, IFN-γ, MCP-1, and TNF-α increased in pristane-injected WT mice but not in *Cgas^-/-^* mice. However, serum IL-1α, not IL-1β, was raised in pristane-injected *Cgas^-/-^* mice. These results suggested that PIL may be activated *via* the caspase-11 noncanonical inflammasome in the lungs of *Cgas^-/-^* mice.

Inflammasome activation can lead to cell death *via* pyroptosis. *In vitro* experiments showed that BMDMs from *Cgas^-/-^* mice generated more dead cells in response to LPS, dAdT, nigericin, and cGAS agonists than WT mice. These data suggested that the cGAS signaling pathway might be involved in pyroptosis in BMDMs. The high MHC class II (or IAb) expression on macrophages indicated the activated M1 macrophage phenotype (58). Interestingly, increased activated macrophages (CD11b^+^F4/80^+^IAb^+^) after stimulation with a cGAS ligand (G3-YSD) in *Cgas^-/-^* mice suggested that G3-YSD could stimulate other DNA sensors, leading to increased activated macrophages. cGAMP can activate the inflammasome through AIM2 and NLRP3 (22). Additionally, IL-1β production decreases with dAdT-activated inflammasomes in *Cgas^-/-^* cells (22).

The overexpressing DNaseI reduces inflammation in chronic pristane-mediated peritonitis (18), suggesting an essential role of dsDNA in the pathogenesis of pristane-induced inflammation. The *Dnase2*-deficient mice showed lethal inflammation and were rescued in the absence of cGAS (8), suggesting that cGAS mediates inflammation in a spontaneous mouse model. This data suggested the link between DNA sensing cGAS and DNase2-mediated DNA degradation. However, cGAS-deficient mice develop severe lupus disease in the pristane-induced mouse model. The different results between the models could cause by the separate compartments of ligands that encounter DNA sensors.

A recent study shows that DNA binding cGAS forms a liquid-liquid phase separation (LLPS) which activates innate immunity (59). Also, the LLPS enhances DNA-activated cGAS signaling (60) and regulates autophagy (61). The process of cGAMP-induced autophagy is essential for the clearance of DNA and viruses in the cytosol (62). The increased dsDNA in the absence of cGAS may be a compensatory mechanism to increase the other DNA sensor’s activation, or cGAS may play a role in DNA clearance through LLPS-mediated autophagy.

The data suggested that the increased intracellular dsDNA in pristane-injected *Cgas^-/-^* mice could activate both AIM2 and NLRP3 inflammasomes, causing cell death and tissue inflammation.

In summary, we demonstrated that *Cgas^-/-^* mice exhibit enhanced autoantibody production and lung inflammation in PIL. The macrophage infiltration in the lung and the activated macrophages in the spleen of *Cgas^-/-^* mice, but not dendritic cells, suggested the differential role of cGAS in a specific cell type. Overall, the cGAS sensor promotes inflammatory responses in the lung in a pristane-induced lupus model. Without cGAS, cytosolic dsDNA could overflow in the cells and activate noncanonical inflammasomes leading to IL-1α production. The cGAS agonists could activate macrophages in the cGAS-independent pathway. These fundamental findings suggested that cGAS hampers the unusual noncanonical inflammasome activation through other DNA sensors, which could be a new targeted therapy for treating autoimmune disease.

## 5 Conclusion

The absence of cGAS leads to the rise of cytosolic dsDNA, accelerates noncanonical inflammasome activation in macrophages, and aggravates lupus pathology in the pristane-induced lupus model.

## Data availability statement

The original contributions presented in the study are included in the article/**Supplementary Material**, Further inquiries can be directed to the corresponding authors.

## Ethics statement

All animal experiments were reviewed and approved by the Institutional Animal Care and Use Committees (IACUC) of the Faculty of Medicine, Chulalongkorn University (019/2562 and 024/2563).

## Author contributions

SK performed the experiments, interpreted the data, codirected the study, and wrote the manuscript. AT-U provided experimental assistance for BMDC. CT provided experimental assistance for flow cytometry. NK and NC performed and analyzed the western blot experiment. AL analyzed and scored the histopathology of tissue sections. TP contributed reagents, designed the experiment, codirected the study, and edited the manuscript. PP performed the experiments, designed the experiments, interpreted the data, directed the studies, and wrote the manuscript. All authors contributed to the article and approved the submitted version.
