# Supplementary figures and images for: cGAS deficiency enhances inflammasome activation in macrophages and inflammatory pathology in pristane-induced lupus
Source: Front Immunol. 2022 Dec 16;13:1010764. doi: 10.3389/fimmu.2022.1010764 (PMC9800982; doi:10.3389/fimmu.2022.1010764)

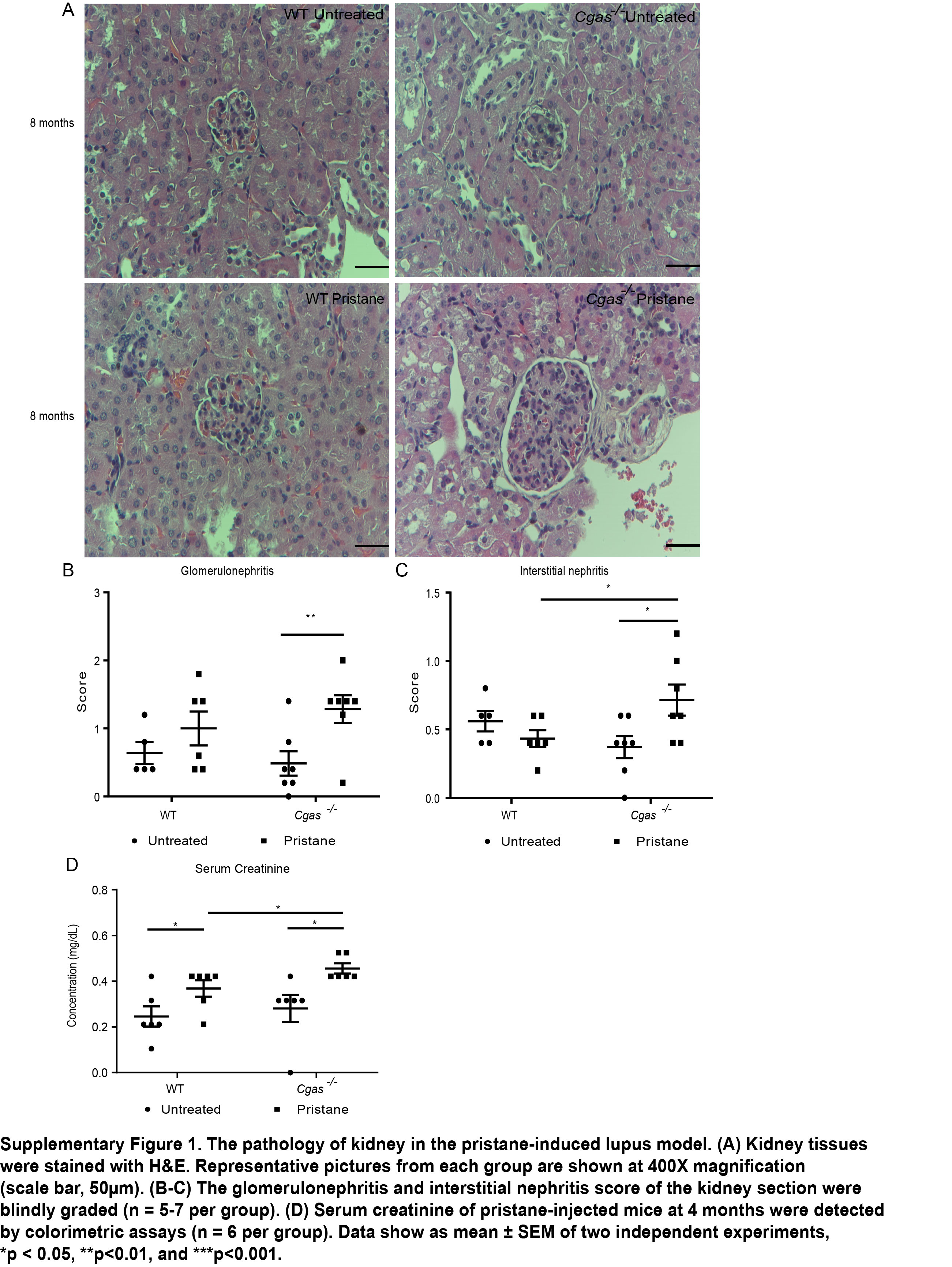

Supplement: Supplementary file 1 [file Image_1.jpeg]

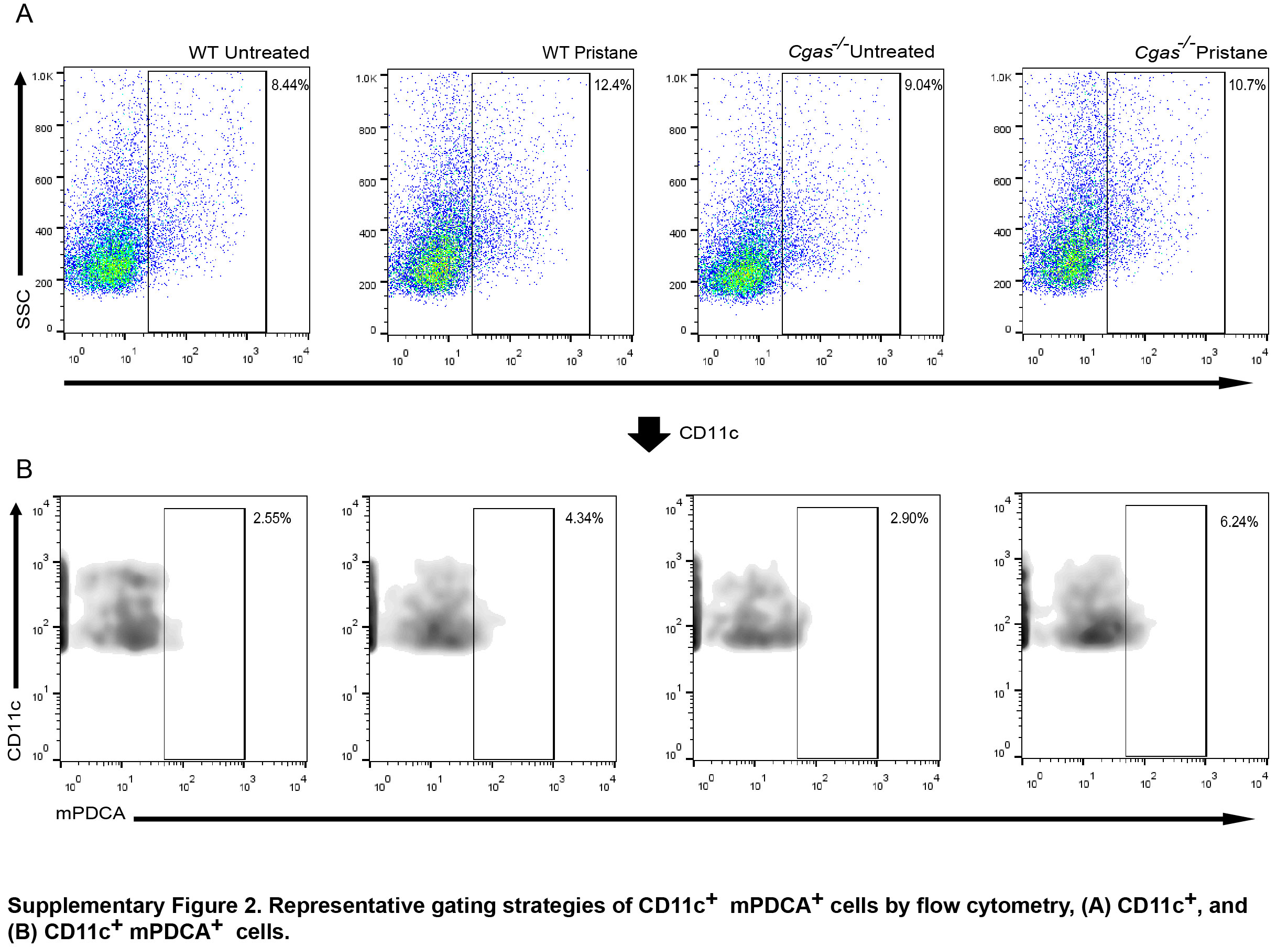

Supplement: Supplementary file 2 [file Image_2.jpeg]

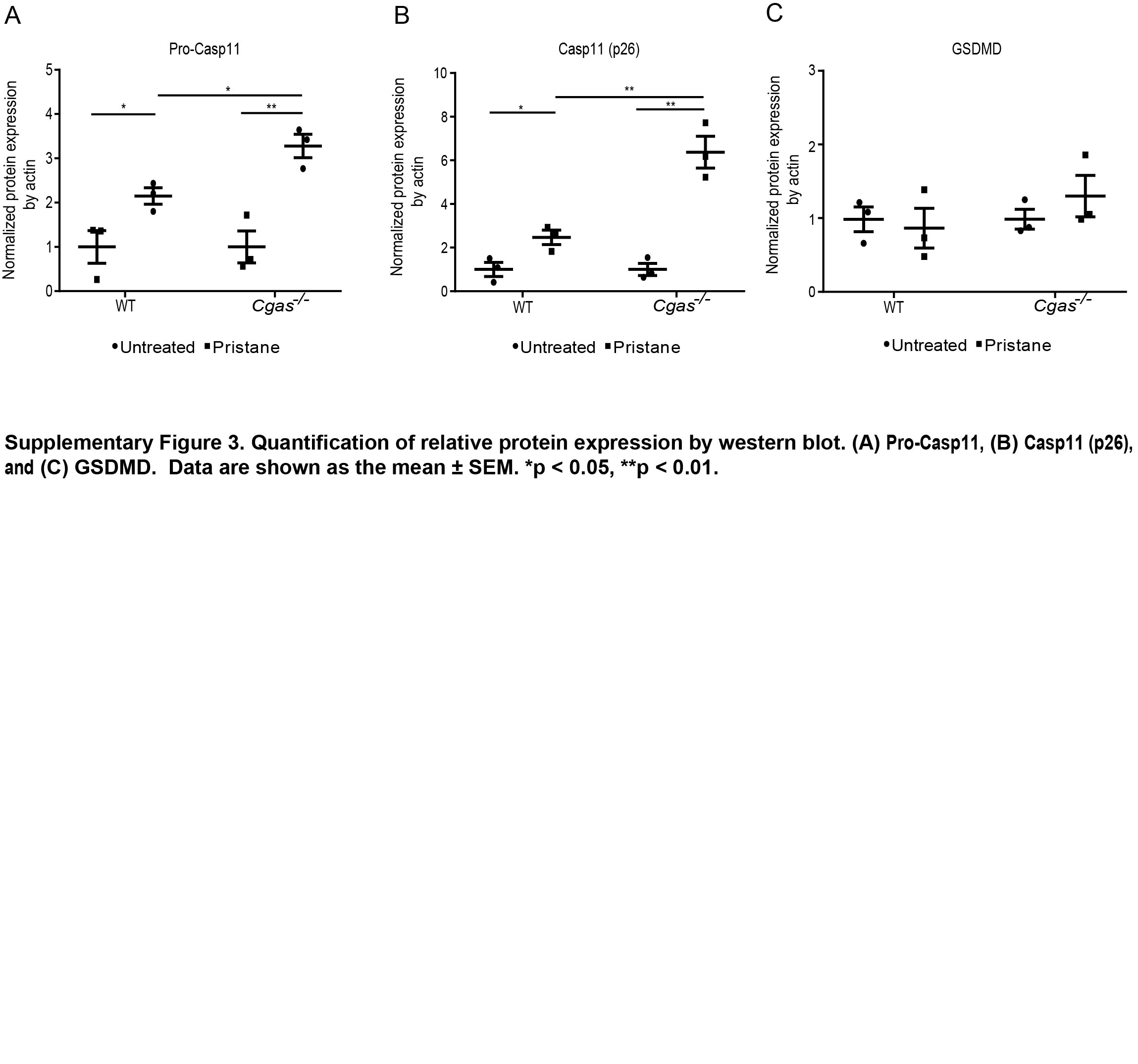

Supplement: Supplementary file 3 [file Image_3.jpeg]

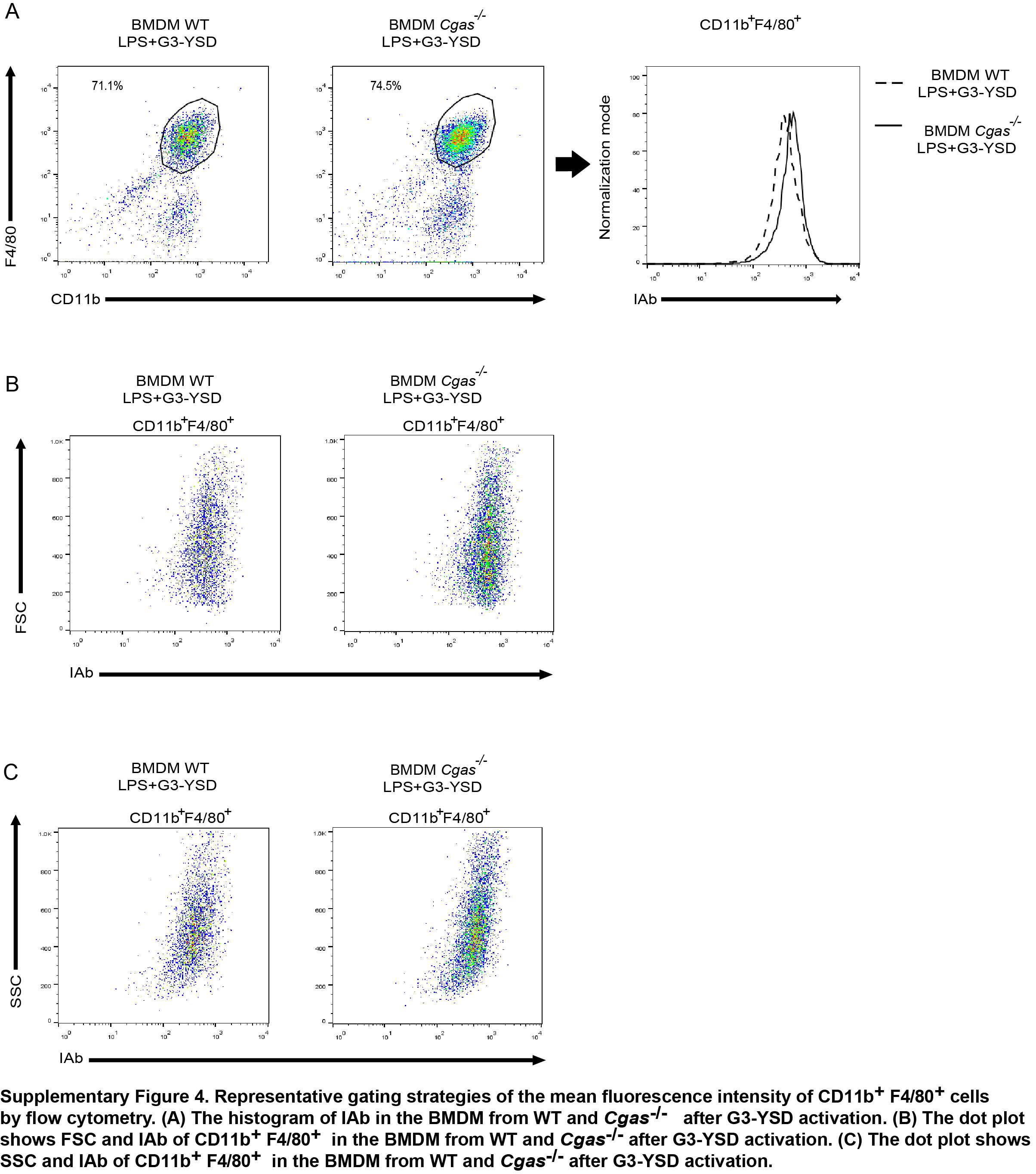

Supplement: Supplementary file 4 [file Image_4.jpeg]
